# Deep Learning-Based LOS and NLOS Identification in Wireless Body Area Networks

**DOI:** 10.3390/s19194229

**Published:** 2019-09-29

**Authors:** Krzysztof K. Cwalina, Piotr Rajchowski, Olga Blaszkiewicz, Alicja Olejniczak, Jaroslaw Sadowski

**Affiliations:** Faculty of Electronics, Telecommunications and Informatics, Gdansk University of Technology, 80-233 Gdansk, Poland; piorajch@eti.pg.edu.pl (P.R.); olga.blaszkiewicz@pg.edu.pl (O.B.); alicja.olejniczak@pg.edu.pl (A.O.); jaroslaw.sadowski@eti.pg.edu.pl (J.S.)

**Keywords:** deep learning, machine learning, UWB, BAN, WBAN, LOS, NLOS, DWM1000, channel impulse response

## Abstract

In this article, the usage of deep learning (DL) in ultra-wideband (UWB) Wireless Body Area Networks (WBANs) is presented. The developed approach, using channel impulse response, allows higher efficiency in identifying the direct visibility conditions between nodes in *off-body* communication with comparison to the methods described in the literature. The effectiveness of the proposed deep feedforward neural network was checked on the basis of the measurement data for dynamic scenarios in an indoor environment. The obtained results clearly prove the validity of the proposed DL approach in the UWB WBANs and high (over 98.6% for most cases) efficiency for LOS and NLOS conditions classification.

## 1. Introduction

Undoubtedly, WBANs (Wireless Body Area Networks) have gained immense popularity due to their use in modern 5G networks, where users expect the quality and speed of data streaming services to be increased while maintaining mobility. Monitoring vital signals in medical surveillance systems, increasing the safety of users or officers during operational tasks, radiolocation in short-range systems or applications related to entertainment are ones of the main applications of these networks.

From the WBANs designing procedure point of view, the influence of the human body has a significant impact on the radio channel characteristics in the communication inside the human body (*in-body*), on the human body (*on-body*), between bodies (*body-to-body*), and between the human body and an external access point (*off-body*) [1].

In this article, the research is focused around the *off-body* communication, which enables the information transmission outside the human body and is the most commonly used type of communication in the RTLS (Real Time Locating Systems). In such systems, operating in an indoor environment, due to, e.g., high time of flight measurement resolution, UWB (Ultra-Wideband) radio interfaces are widely used. They are characterized also by a higher robustness to the multipath propagation effect than narrowband radio interfaces and allow obtaining even centimeter localization accuracy [2,3]. One such radio interface is the well-known DecaWave DWM1000 radio module, which was also used in this study [4].

Currently published research works are aimed at increasing the efficiency of radiocommunication systems and networks, e.g., by dynamically determining the conditions of nodes direct visibility LOS (Line-of-Sight) or the lack of direct visibility NLOS (Non-Line-of-Sight) [2,5,6]. The high efficiency of methods for detecting such conditions can significantly increase the quality of offered services, e.g., by compensating the increasing error of radio distance measurements in variable propagation conditions or by dynamically changing the transmission bitrate to minimize the packet error rate in the network [2].

The dynamic development of an AI (Artificial Intelligence), including ML (Machine Learning) and DL (Deep Learning), mainly in the field of informatics, automation and robotics also increases interest in the usage of DL methods in the radiocommunication and NGNs (Next Generation Networks) [7,8]. DL enables solving multidimensional optimization problems and allows not only adaptive self-learning of complex, non-linear relations, or features of a given dataset, but also prediction of their values [9]. Considering this, the DL application in the deep, multilayer neural network DNN (Deep Neural Network) form can also be successfully used as a method for determining LOS and NLOS conditions in UWB WBANs.

The main goal of this research is to properly design a DNN that will allow achieving greater classification efficiency, i.e., accuracy metric understood as the ratio of the correctly classified data to the total number of performed classification [9], in these conditions with comparison to existing solutions. Thus, DNN architecture direct parameters estimation, including the input data type from CIR (Channel Impulse Response), which do not require more complex architecture, e.g., including convolutional layers, was developed. Furthermore, it can be implemented in practical applications, and even on microcontrollers with reduced computational power.

## 2. Related Work

Currently, various methods for identifying LOS and NLOS conditions in UWB networks are known in the literature, including solutions based on ML, i.e., SVM (Support Vector Machine). In general, these methods can be divided into those that uniquely determine LOS and NLOS conditions based on current parameters in real time [5,6,10,11] or those that have a certain time inertia, e.g., due to the additional data filtration usage and data processing with memory [2]. In this article, only real-time working methods will be analyzed.

The producer of the mentioned DWM1000 radio module, in [4], presented the threshold method (THM) for detecting NLOS conditions, based on the difference between the estimated total received signal power *TP* (Total Power) [dBm] and the power of the first received component *FPP* (First Path Power) [dBm] that is possible to obtain directly from the module registers, according to the formula.

(1) TFD [dB] = TP [dBm] − FPP [dBm],

where *TFD* [dB] is a metric specifying the conditions of direct visibility and according to [4] should take values greater than 10 dB for NLOS conditions.

In [10], authors presented a new, two-stage SVM classification method, which allows the LOS and NLOS identification with the existence of the main received signal component distinction. Stationary measurements were performed in the indoor environment using DWM1000 modules and the obtained results were subjected to the ML algorithm. Based on the results, authors obtained 93.7% efficiency in detecting LOS and NLOS conditions, where the SVM input data were different combinations of extracted features of the estimated CIR, e.g., mean value, variance, or the channel impulse response delay spread.

In [6] the method of identifying NLOS conditions and the use of regression method to reduce the mean square localization errors in these conditions are presented. Authors also applied the extracted features of the estimated radio CIR and the obtained 91% classification efficiency for static scenarios contributed to the increase in the localization accuracy of UWB localization system.

Similar NLOS conditions detection research is also conducted in WBANs, where the human body may obstruct the direct visibility of nodes. In [11], the authors obtained 87.5% classification efficiency for *on-body* and *off-body* communication by reducing the full CIR by PCA (Principal Component Analysis) used as an input for the SVM method.

Further research is presented in [5], where authors performed studies for both static and dynamic scenarios by comparing the THM described by the DWM1000 modem manufacturer with the MLP (Multi-Layer Perceptron) method proposed by the authors, with a total of 20 nodes, and a reinforced tree BTD (Boosted Decision Trees) method. The authors obtained 47% to 98% efficiency for THM, depending on the selected threshold value and 98% for MLP and BDT methods for static scenarios. However, the results of dynamic scenarios analysis showed a significant decrease, in relation to static scenarios, in the effectiveness of the analyzed methods, i.e., from 64% to 91% for THM, 92% for the MLP, and 94% for the BDT method in a laboratory environment, and from 70% to 79% for THM, 82% for the MLP, and 87% for the BDT methods in a factory environment with a strong multipath propagation effect.

## 3. Proposed Deep Learning Approach

Taking into consideration methods proposed in the literature and their limited efficiency, it was decided to develop a DNN that, comparing to threshold and ML methods, will achieve higher efficiency in identifying LOS and NLOS conditions for dynamically moving users in an indoor environment with enhanced multipath propagation effect. Compared with the obtained DL method, for ML methods presented in the literature many input CIR features are manually selected and analyzed, thus the dependencies between the basic CIR parameters were also enforced. Proposed DNN method needs only limited input parameters for higher, than in literature, classification efficiency. Furthermore, the proposed DNN can learn single-handedly the non-linear dependencies to maximize the LOS and NLOS conditions classification effectiveness.

**Figure 1:**
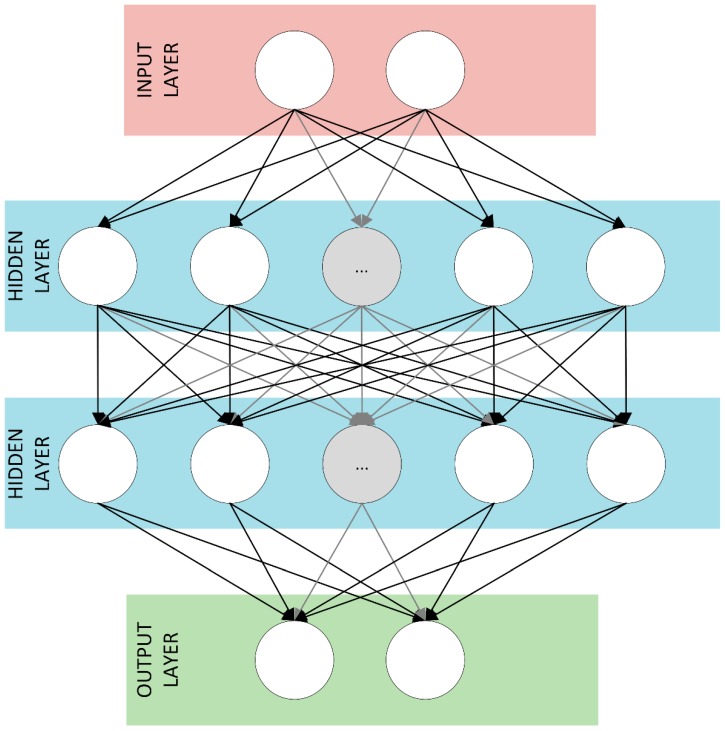
Architecture of the proposed Deep Feedforward Neural Network (DFNN).

It was assumed that the full CIR will not be analyzed together with a combination of several distinguished features from it. Basing on only one CIR parameter, e.g., received signal strength estimation, is not sufficient because of the very limited applicability of the established method—it would be limited to a specific scenario and vulnerable to dynamic propagation condition changes. Still, in some practical applications, methods requiring only one input parameter are efficiently used [12]. Thus, the pair of parameters is the minimum number to develop more versatile solutions in described WBANs application. Furthermore, in the proposed DNN only two CIR parameters, which are estimated in the DWM1000 module and read from its registers after the packet has been correctly received, i.e., total power (TP) and the power of the first component (FPP) are analyzed. This assumes a significant simplification of the network structure and reduces the computational effort required for their use in real-time systems. Thus, due to the limited size of the input layer, it was decided to use the Deep Feedforward Neural Network (DFNN), where all layers are fully connected. Detailed mathematical description of the DFNN can be found in [9]. As the proposed DFNN does not have a feedback element, each decision about the LOS and NLOS conditions is made independently, just based on the pair of CIR parameters, i.e., TP and FPP, from only a single radio packet reception. In Figure 1 the proposed architecture of the developed DFNN is presented.

Usually, in the DNN algorithms for determining the optimal parameters of these networks without having to check all combinations are implemented, e.g., using genetic algorithms, random search, or grid search [13,14]. Considering the proposed architecture of the DFNN and its limited number of parameters to check, relative to, e.g., image processing convolutional networks, it was decided to test their combinations without using additional algorithms that search in the space of possible solutions.

Initial weights were generated with uniform distribution using the method described in [15]. Based on the research, it was decided to use two hidden layers, where the number of nodes in each layer was 50. It is worth noting that a further increase, e.g., in the number of hidden layers or in the number of nodes in individual layers, resulted in a network efficiency increase by an average value of 0.05% for all combinations of training sets, which was not satisfactory in relation to significant increase in the number of calculations to apply more extensive architecture. In Table 1, the example results of the classification efficiency in relation to the chosen two hidden layers as a function of hidden layers number are presented. The results for each hidden layer configuration are mean values of the obtained classification efficiency for changeable number of nodes in the range of 25–200.

In order to make a non-linear description of the input data features in the neural network model, a non-linear activation function was used, i.e., a sigmoidal function for all hidden layers. In the output layer, the LogSoftmax activation function is calculated, whose normalized output value corresponds to the estimated probability of belonging a given input dataset to a selected class, i.e., LOS or NLOS [9].

The selected structure of the DFNN determines the number of mathematical operations (especially multiplication operations) that must be performed to carry out the learning process, and then usage of the developed model in real-time systems. The computational complexity of unidirectional networks without feedback depends on the number of layers and nodes, as well as the used activation function, which should be treated directly as matrix operations [9,16]. These operations can be parallelly calculated, e.g., through multi-core processors or graphics cards, which significantly speeds up the network learning phase.

For the proposed architecture, to determine LOS and NLOS conditions basing on a single pair of CIR parameter values, 2700 multiplication operations and the same number of summations are required. These numbers do not include calculations related to non-linear activation functions. In [17], an analysis of the computational complexity of these functions is presented, which, depending on the accuracy required, can be approximated by linear functions or read from fixed array. Bearing this in mind, the proposed DL method can be successfully used in real-time systems, even on microcontrollers with hardware support of floating-point operations.

The quality of the network learning process was monitored by a NLLL (Negative Log Likelihood Loss) function, which minimization shows the learning process positive effect [9]. Based on the loss function value, the ADAM (Adaptive Moment Estimation) algorithm with selected parameters (α = 0.001, $\beta_{1}=\;0.9$, $\beta_{2}=\;0.999$, and ${\varepsilon \;=10}^{-8}$) was used to optimize the learning process. The ADAM algorithm is described in detail in [18]. In general, it is a modified classic SGD (Stochastic Gradient Descent) algorithm which was chosen due to the computational efficiency and faster obtained model convergence, comparing to other algorithms. It is achieved by the adaptive learning factor based on the calculated first and second gradients moments during the nodes weight updating process (the learning process) [18].

For the learning process, each time the weights are updated for backpropagation process a greater number of mathematical operations should be considered relative to the already learned model. This is due to, among others, from the need to calculate the gradient of the used activation functions [9]. The number of weight numbers (and thus the computational complexity of the learning process) is related to the method used to optimize the learning process, the method of data fragmentation, and the final number of iterations required to achieve learning process convergence, which is strongly dependent on the analyzed datasets. However, it should be remembered that the learning process—despite greater computational complexity—is carried out once and does not limit the possibility of working the proposed method in real-time systems.

## 4. Measurement Scenarios

**Figure 2:**
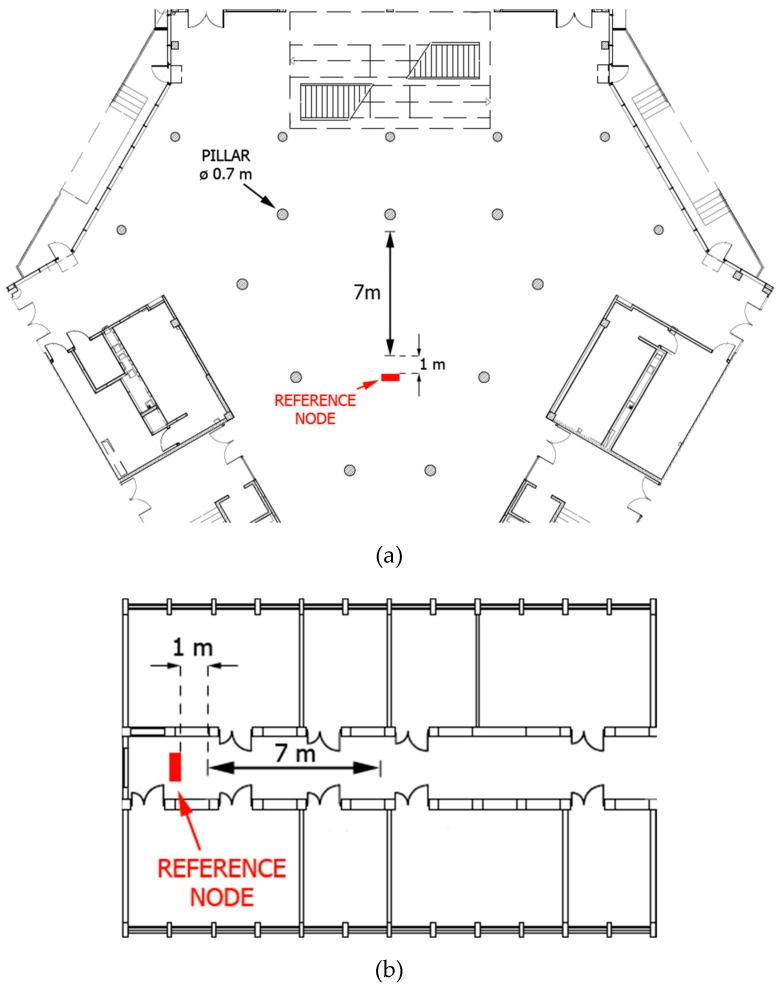
Two-dimensional plans of the indoor measurement environments: (**a**) hall, (**b**) corridor.

Most of the currently designed wearable radio networks find their application in environments with disturbed radio waves propagation, where the multipath propagation effect is strongly emphasized. This has a large impact on the effectiveness of methods for determining the direct visibility conditions between nodes and the interpretation of the CIR. For this reason, it was decided to implement two dynamic measurement scenarios (S1, S2) for *off-body* communication in a typical indoor environment. Both scenarios were conducted respectively in the hall (S1) and the corridor (S2) of the Faculty of Electronics, Telecommunications, and Informatics of the Gdansk University of Technology. In Figure 2, the two-dimensional plans of the both indoor environments are presented.

All scenarios were conducted by attaching the stationary reference node (RN) to a tripod at a 1.2 m height, and the mobile node (MN) on the body of a moving person on the chest (TO) or at the front side of the waist (WS). The separation between human skin and radiating elements was about 1 cm.

The nodes contain the DecaWave DWM1000 radio module, operating at 6489 MHz, with a 499.2 MHz bandwidth, where the transmission speed in the radio channel was 6.8 Mb/s. Selected radio module is compliant with the IEEE 802.15.4-2011 standard [4]. A detailed description of the developed mobile measurement stand can be found in [2,19,20].

The measurements were carried out by three women (W1, W2, W3) and two men (M1, M2) of different weight and height. It is worth noting, that these people participated in selected measurement scenarios to be able to check the proposed DL method effectiveness in completely different conditions. In the Table 2 selected parameters of people participating in the measurements, i.e., Body Mass Indexes (BMIs), heights $h_{MM}$ of wearable device montage placements, and realized measurement scenarios, are presented.

Most of the currently presented research results of methods effectiveness for determining NLOS conditions include results mainly from static scenarios. However, dynamic scenario studies are characterized by significantly lower classification efficiency due to variable propagation conditions [5]. Bearing this in mind, it was decided to carry out measurements for dynamic scenarios, which, due to the occurrence of the fast fading component associated with the change in body position and human movement, should be treated as more difficult one in terms of radio wave propagation compared to static scenarios [2,19,20,21].

The measurements were carried out for the scenarios of approaching and departing, relative to the RN, of people with mounted MN. The LOS and NLOS conditions were distinguishable by the human body occurrence between the pair of nodes, i.e., the approaching scenario is classified and labeled as LOS conditions and the departing scenario as NLOS conditions. Antennas orientation were matched, thus QLOS (Quasi Line-of-Sight) conditions did not occur [21], in provided measurement scenarios. Each person walked along the marked route in a straight line at a distance of 7 m, i.e., from 1 m to 8 m with relation to stationary RN. Each person made 20 repetitions for both approaching and departing scenarios. The selected CIR parameters, i.e., TP and FPP, were estimated with a 25 Hz frequency.

## 5. Analysis of the Results

During the measurements, over 40,000 pairs of CIR parameter values were obtained for all measurement scenarios. It was decided to carry out classification effectiveness *η* measurements of the proposed DFNN, THM, and SVM methods, which were widely used in the studies presented in the literature [5,6,10,11]. It should be mentioned that the SVM method was also tested with the use of only two input parameters, i.e., FPP and TP. Training and testing datasets were fully disjoint, i.e., testing sets were the results of data for people other than the training sets, e.g., for the one-person training set it was the results of measurements for person W1, while the testing sets were the results of measurements for all the other persons W2, W3, M1, and M2. Similar to the one-person datasets, the two-person datasets were also fully disjoint, e.g., if the training set was the results of measurements for people W1 and W2, the testing sets were the results of measurements for all the other persons W3, M1, and M2 respectively. The network was trained for 100 full iterations of the training set with 64 batches segmentation used to update the weights of the developed DFNN. For results reliability, both the LOS and NLOS datasets had exactly the same quantity.

Firstly, the possibility of identifying LOS and NLOS conditions based on a one-person learning dataset was checked. In Table 3, the effectiveness of the proposed DL method and other methods are presented.

The proposed DL method obtained the best results for all analyzed datasets. For some persons, it is possible to achieve the classification effectiveness definitely greater (reaching even 99.6%) than the simple THM, which should be treated as a reference method, i.e., the lower bound of acceptable performance values, or the SVM method, for which the values for some datasets were worse than THM.

It is worth noting that the presented results of the THM are obtained for a dynamically variable TDF metric, in the range of 2 dB to 10 dB, depending on the training dataset, which differed in relation to individual scenarios by up to 3 dB. For the 10-dB threshold value proposed in [4], the THM efficiency did not exceed 60%.

**Figure 3:**
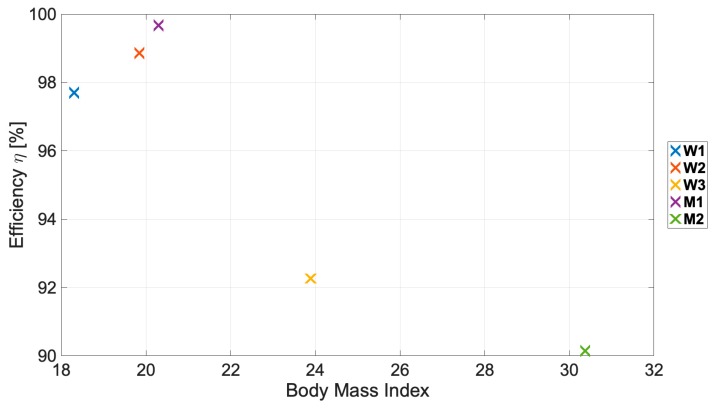
DFNN effectiveness *η* as a function of the Body Mass Index (BMI) parameter for the one-person training set.

The obtained results were analyzed by taking into account the impact of the human body structure on classification effectiveness. In Figure 3, a graph of the developed DFNN effectiveness as a function of the BMI parameter for the one-person training set is presented.

Based on the obtained results, it could be seen that the body characteristics, including, e.g., fat and muscle tissues, have higher impact on the obtained classification effectiveness than analyzed environment structure. For the conducted measurements, the proportional dependence of the classification efficiency and the BMI is visible, regardless of the indoor environment structure (hall, corridor) or the height of montage placements because of the body structures, i.e., fat and muscle tissue ratio and the way of calculating the BMI parameter.

A significant decrease of the classification efficiency can be seen between the M1, W3, and M2 one-person training datasets respectively. Mainly, this is due to the difference in body structure. Woman W3 had higher muscle tissue relative to fat comparing with the M1 man, who is only 4 cm shorter but almost 14 kg lighter and had lower muscle and fat tissues. A similar situation can be seen between the W3 and M2 pair, where person W3 is almost 20 kg heavier (mainly due to much higher fat tissues), with only a 1-cm difference in height.

Considering the above, irrespective of the measurement scenario it is possible to classify LOS and NLOS conditions on the basis of even a single-person learning dataset with satisfactory accuracy (even above 97%) for dynamic scenarios, which is a higher value than those presented in the literature [5]. This simplifies the process of designing UWB WBANs, because even a single-person measurement series and the proposed DFNN method can allow obtaining a high probability of correct classification of node visibility conditions during system operation, even for different structures of the indoor environment. However, a one-person training dataset should be based on data for a person with the lowest BMI indicator, so that the network is sensitive to changes caused by the human body shadowing the direct visibility conditions of WBAN nodes.

**Figure 4:**
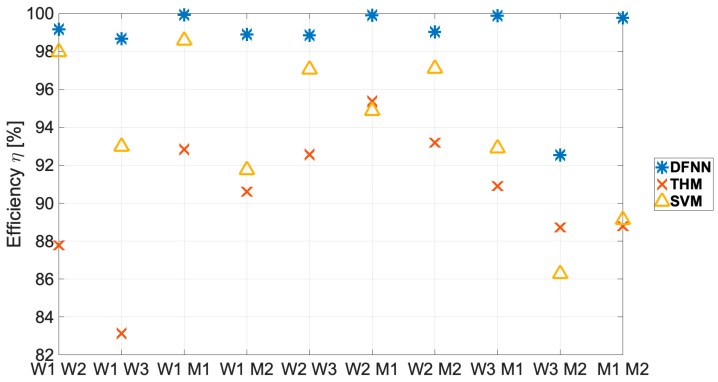
Selected methods effectiveness *η* for two-person training sets.

Then, the possibility of estimating LOS and NLOS conditions based on a two-person training dataset was evaluated. In Figure 4, a graph of selected methods effectiveness *η* for two-person training sets is presented.

For all analyzed two-person training datasets combinations, the proposed DFNN method achieved the highest results, with a median detection efficiency of 99.1%, a maximum of 99.8%, and only one value was below the 98.6% threshold. The smallest classification value corresponds to the scenario for persons W3 and M2, who have the highest BMI values, which corresponds to the classification results presented in Figure 3 for one-person training datasets.

As expected, in most cases the lowest efficiency was obtained for THM method. However, both the THM and SVM are characterized by a visible instability of convergence with respect to individual learning datasets. This results in a greater or similar effectiveness of the THM relative to SVM for several learning datasets, which in these cases calls into question the legitimacy of using the more complex SVM method. The obtained values of the THM classification efficiency and SVM methods are similar to these presented in the literature [5,6,10,11]—described in more detail in Section 2.

There is also a visible decrease in the variance of the DFNN effectiveness results for a two-person training dataset and compensation of the negative body impact with a higher BMI on the quality of the classification relative to a one-person training dataset. In practice, this means that the performance of a measurement series for two people and the use of the proposed DNN enables repeatable high efficiency in identifying LOS and NLOS conditions in WBANs using only two values of the estimated CIR without any additional delay.

## 6. Conclusions

Identifying the direct node visibility conditions in radio networks is an important issue, especially in the context of radiolocation systems or networks with dynamically allocated resources. The growing interest in aspects of AI, including DL and their usefulness, e.g., in solving optimization problems, determines the adaptation of self-learning methods also in radiocommunication systems.

In the article, the use of DL in UWB WBANs, which allows achieving high efficiency in determining the LOS and NLOS conditions for dynamic scenarios *off-body* communication, is presented. The proposed DFNN method needs only two input parameters for sufficient (over 98.6% for most cases) classification efficiency and can learn the non-linear dependencies between these two parameters to maximize the classification effectiveness. Based on the research, two hidden layers, where each consists of 50 nodes, were estimated as an optimal DFNN architecture to be used in the considered application. Thanks to this, the proposed method can be implemented in a wearable node based on integrated microcontrollers and operate in a real time. It was determined that for obtained measurements the body structure of people participating in the learning process had a greater impact on the obtained classification results than environment structure.

It is possible to achieve classification efficiency over 97% already for the one-person learning dataset, assuming that this person will have a relatively low BMI in relation to the group of persons using a given network. Gain resulting from the usage of the proposed DNN increased with the enlargement of the training dataset to two persons, and the obtained classification efficiency for individual training datasets, in almost all cases, exceeded the value of 98.6%. In summary, based on the provided research the training dataset should include the data for persons with a low BMI, arising from low fat and muscle tissues, so that the network is sensitive to changes caused by the human body shadowing the direct visibility conditions of the WBAN nodes.

The presented results predispose the proposed DFNN for use in real-time WBANs. Based on two parameters obtained from the CIR treated as an input data to the DFNN enables high operational efficiency based on a limited measurement series for one or two persons training datasets, regardless of the indoor environment structure.

Future works will be focused on executing more measurements in several different indoor and outdoor environments with various persons, to check the dependence of the body and environment structure on the DNN efficiency in a larger dataset. In addition, future measurements will include more detailed analysis of body structure, e.g., fat and muscle tissues, to give more information rather than using a simplified BMI indicator. Based on the obtained empirical data, an attempt to improve the quality of detection, by taking into account the history of decisions in the DNN with memory, will be also performed.

## Figures and Tables

**Table 1:** Results of the classification efficiency.

Number of Hidden Layers	2	3	4	5	>5
Classification efficiency [%]	98.66	98.70	98.71	98.72	98.72

**Table 2:** Selected parameters of people participating in the measurements.

	Height (m)	Weight (kg)	BMI	Montage	$h_{\mathrm{MM}}\;(m)$	Scenario
W1	1.75	56	18.3	WS	1.10	S1
W2	1.68	56	19.8	WS	1.10	S1
W3	1.76	74	23.9	WS	1.10	S1
M1	1.72	60	20.3	TO	1.35	S2
M2	1.75	93	30.4	TO	1.35	S2
W – Woman, M – Man; BMI – Body Mass Index; WS – Waist; TO – Chest; S – Scenario;						

**Table 3:** Classification effectiveness *η* results of selected methods for one-person training set.

		W1	W2	W3	M1	M2
η (%)	**DFNN**	**97.7**	**98.8**	**92.2**	**99.6**	**90.1**
	SVM	93.9	97.8	87.8	95.8	86.4
	THM	86.0	93.9	90.1	90.9	89.2
